# Identifying combined design and analysis procedures in two-stage trials with a binary end point

**DOI:** 10.1002/sim.5468

**Published:** 2012-07-11

**Authors:** Jack Bowden, James Wason

**Affiliations:** MRC Biostatistics UnitCambridge, U.K.

**Keywords:** optimal design, admissible design, bias-adjusted estimators, mean squared error

## Abstract

Two-stage trial designs provide the flexibility to stop early for efficacy or futility and are popular because they have a smaller sample size on average than a traditional trial has with the same type I and II error rates. This makes them financially attractive but also has the ethical benefit of reducing, in the long run, the number of patients who are given ineffective treatments. Designs that minimise the expected sample size are often referred to as ‘optimal’. However, two-stage designs can impart a substantial bias into the parameter estimate at the end of the trial. In this paper, we argue that the expected performance of one's chosen estimation method should also be considered when deciding on a two-stage trial design. We review the properties of standard and bias-adjusted maximum likelihood estimators as well as mean and median unbiased estimators. We then identify optimal two-stage design and analysis procedures that balance projected sample size considerations with those of estimator performance. We make available software to implement this new methodology. Copyright © 2012 John Wiley & Sons, Ltd.

## 1. Introduction

When investigating new and potentially promising treatments in the early stages of drug development, it is common to conduct a small-scale single-arm trial. The outcome is often defined simply in terms of a binary response (e.g. success or failure). The success probability of the current standard treatment is usually assumed to be known, *p*_0_ say, so that the experimental treatment may warrant further investigation if it has a success probability *p* such that *p*⩾*p*_1_ > *p*_0_, where *p*_1_ represents the smallest clinically relevant improvement. Two-stage designs that allow the possibility to stop early for efficacy or futility are a popular choice for such trials because they have a smaller sample size on average compared with a traditional trial with the same type I and II error rates. This makes them financially and ethically attractive. Although the main purpose of such a trial is to make a decision on whether to stop or continue research into the experimental treatment, accurate estimation of its success probability is still important to appropriately power future studies. However, two-stage designs can impart a substantial bias into the parameter estimate for *p*. As we will show, this is especially true for designs that minimise the expected sample size.

In this paper, we review and evaluate several methods for estimating the response probability in a two-stage trial, concentrating on their bias and mean squared error (MSE). This comparison is greatly aided by applying the sample space ‘*T*-mapping’ approach proposed by Jovic and Whitehead [1]. In light of these results, we propose a modification to the admissible design method described in Jung *et al.* [2] and Mander *et al.* [3]. Given desired type I and II error rates as well as prior beliefs about the likely value of *p*, our modification provides a tool enabling the user to identify two-stage design and analysis procedures that balance sample size considerations against the desire for accurate estimation. By incorporating estimator accuracy into the decision framework governing design choice, our approach is similar in spirit to that of Liu [4] but is substantially different in terms of its details and scope. In Section 2, we introduce the *T*-mapping procedure and use this as a common framework for exploring five distinct estimators for *p*. In Section 3, we explore their bias and MSE across the design space of some common two-stage trials. In Section 4, we introduce our algorithm for choosing an optimal design and analysis procedure and demonstrate its use. We conclude with a discussion in Section 5.

### 1.1. Notation

Following the notation in [1], let *X*_*i*_ = (0,1) denote the failure or success of person *i* in a two-stage trial of *n*_2_ people, for which *n*_1_ patients are recruited at stage 1. If 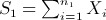 is ⩽*l*_1_, then the trial is stopped at stage 1 for futility (and acceptance of *H*_0_), and if *S*_1_ is ⩾*u*_1_, the trial is stopped at stage 1 for efficacy (and rejection of *H*_0_). If *l*_1_ + 1⩽*S*_1_⩽*u*_1_ − 1, then the trial recruits a further *n*_2_ − *n*_1_ patients leading to a total sample size of *n*_2_. Finally, if 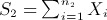 is ⩾*u*_2_, then *H*_0_ can be rejected at stage 2. For trials that allow stopping at stage 1 for efficacy and futility, the values of *n*_1_,*n*_2_,*l*_1_,*u*_1_ and *u*_2_ can be chosen to satisfy the desired type I and II error probabilities. Define 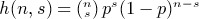 as the binomial probability of observing *s* responses out of *n* given *p*, and let 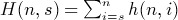.

**Figure 1:**
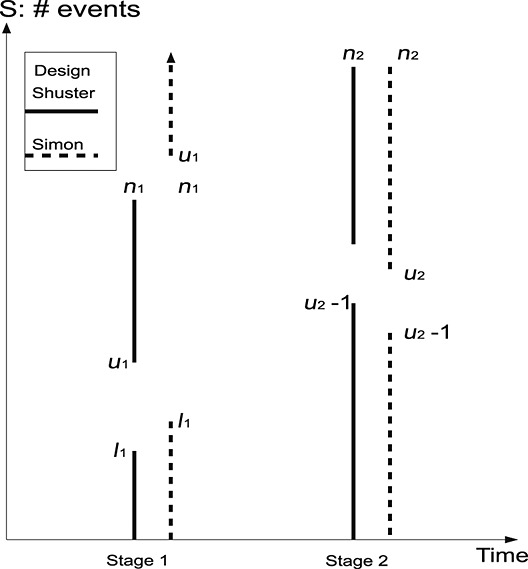
The two-stage design, with stopping efficacy and futility (Shuster-type) and futility only (Simon-type).

All subsequent formulae will assume a two-stage design allowing stopping for efficacy and futility—we will refer to this generically as a ‘Shuster-type’ design [5]. If early stopping for efficacy is deemed inappropriate, we can easily modify these formulae by fixing *u*_1_ to be any integer value greater than *n*_1_, *n*_1_ + 1 say, and noting that (assuming *n* is a positive integer) *h*(*n*,*v*) is zero whenever *v* is negative or greater than *n*. We will refer to a trial of this sort as a ‘Simon-type’ design [6]. We illustrate these two classes of designs in Figure 1, and we will consider both types in this paper.

## 2. Estimating *p*: a review of methods

It is necessary for subsequent development to briefly consider hypothesis testing within the context of a two-stage trial. We can transfer the standard definition of a *p*-value to this setting using a *p*-value function. This expresses, at the point the trial ends, the probability of seeing even more extreme evidence against the null hypothesis than that observed and requires a methodology for ordering the design space. For a Shuster-type design, we can define the Fairbanks and Madsen (FM) ordering [1, 7] as follows. If after *n*_1_ observations the number of responses, *s*_1_, leads to the trial stopping (for efficacy or futility), then the only way that the trial could have produced evidence at least as strong against *H*_0_ would be if ⩾*s*_1_ responses had been observed. However, if after *n*_1_ observations the value of *s*_1_ leads to a continuation to stage 2—and a total of *s*_2_ responses were observed among the *n*_2_ subjects, then there are two possible ways of observing equal or more extreme evidence. The trial could have stopped at stage 1 if *s*_1_ had been ⩾*u*_1_. Alternatively, the trial could have proceeded to stage 2 with any possible value of *s*_1_ as long as ⩾*s*_2_ responses were eventually observed. Thus, we can specify the *p*-value function given the final stage *M* = {1,2}and final response number *S* = {0, …, *n*_2_}. Jovic and Whitehead map the pair *M*,*S* to a single statistic *T* as follows:


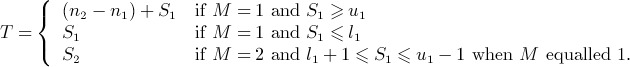
(1)

*T* can take any value between 0 and *n*_2_. As *T* increases, so too does the strength of evidence against *H*_0_ under the FM ordering.

### 2.1. The median unbiased estimate

Jovic and Whitehead utilise *T*-mapping to express the *p*-value function, *K*(*t*,*p*), for the observed value of *T* = *t* and true parameter value *p* as follows:


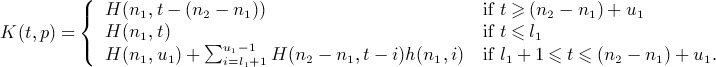
(2)

(also as shown in Table I). They use it as a basis for calculating a fiducial estimate for *p* that is approximately median unbiased. This is achieved by finding a *p*^+^ : *K*(*t*,*p*^+^ ) = 0.5, a *p*^−^ : *K*(*t* + 1,*p*^−^ ) = 0.5 and taking the average: 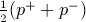. We will refer to this as the median unbiased estimate (MUE). Although not explored in this paper, the same approach is used to find conservative lower and upper confidence bounds for *p* by setting *K*(*t*,*p*) and *K*(*t* + 1,*p*) to *α* ∕ 2 and 1 − *α* ∕ 2, respectively.

**Table I:** *T*-maps for all quantities introduced in Sections 2–4

	Value		
	
Quantity	*t*⩾(*n*_2_ − *n*_1_) + *u*_1_	*t*⩽*l*_1_	*l*_1_ + 1⩽*t*⩽(*n*_2_ − *n*_1_) + *u*_1_
*K*(*t*,*p*)	*H*(*n*_1_,*t* − (*n*_2_ − *n*_1_))	*H*(*n*_1_,*t*)	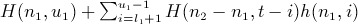
MLE	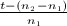	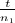	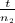
UMVUE	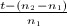	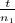	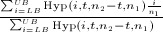
UMVCUE	–	–	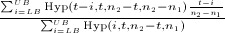
*P*(*T* = *t* | *p*)	*h*(*n*_1_,*t* − (*n*_2_ − *n*_1_))	*h*(*n*_1_,*t*)	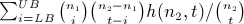
*P*(*T* = *t* | *p*,*l*_1_ + 1⩽*t*⩽(*n*_2_ − *n*_1_) + *u*_1_)	–	–	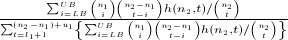

*LB* = max(*l*_1_ + 1,*t* − (*n*_2_ − *n*_1_)) and *UB* = min(*t*,*u*_1_ − 1). 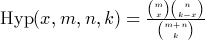.

We now introduce several further estimators for the parameter *p* and express them using the *T*-mapping approach. This helps to facilitate a clearer understanding of their relative utility for two-stage designs explored in later sections.

### 2.2. The maximum likelihood estimate and bias-corrected maximum likelihood estimate

The maximum likelihood estimate (MLE) for *p*, 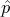, is *S*_1_ ∕ *n*_1_ when *M* = 1 and *S*_2_ ∕ *n*_2_ when *M* = 2. This can be expressed using *T*-mapping as shown in Table I. The method of maximum likelihood does not attempt to address the issue of bias induced by the two-stage design. In this context, Jung and Kim [8] show that


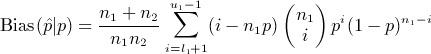
(3)

So, the bias in the MLE is a function of the true value of the parameter *p*, the stage 1 and 2 sample sizes and the bounds for proceeding to stage 2. As a general solution to this problem, Whitehead [9] proposed a bias-corrected maximum likelihood estimator 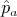 defined via the following relation:


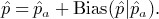


The bias-corrected approach can be applied to this setting using the MLE's *T*-map and Equation (3). We will refer to it as the bias-corrected MLE (BC-MLE).

### 2.3. The uniformly minimum-variance unbiased estimator and uniformly minimum-variance conditionally unbiased estimator

Jung and Kim [8] show that, when evaluating a binary response in a multi-stage trial, the pair (*M*,*S*) are jointly complete and sufficient statistics for the parameter *p*. They then use the Rao–Blackwell theorem to derive its uniform minimum variance unbiased estimate (UMVUE) by calculating 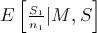, the expected value of the unbiased stage 1 estimate for *p*, given *M* and *S*. We will explore its utility in the two-stage trial setting only. When *M* = 1, the UMVUE is simply equal to *S*_1_ ∕ *n*_1_. When *M* = 2, it is the expected value of *S*_1_ ∕ *n*_1_ given that *S*_2_ patients responded in total and *S*_1_ was in the stage 1 continuation region. This can be intuitively expressed using hypergeometric probabilities and is shown this way (via *T*-mapping) in Table I.

Pepe *et al.* [10] argue that in the context of two-stage Simon-type trial, the estimate for *p* is only of real consequence when the trial continues to stage 2, as it is then used to plan future studies. They therefore suggest the use of the estimator:


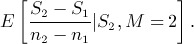


Given *M* = 2, the second stage estimate is unbiased for *p*, and *S*_2_ is its complete sufficient statistic. It is thus the UMVUE conditional on getting to stage 2. We refer to it as the UMVCUE and express it using *T*-mapping in Table I. Note that the UMVCUE is very similar in form to the UMVUE at stage 2, but it differs because the expectation is taken with respect to the second stage estimate and not the first. This clarifies that whereas the UMVUE is unbiased over all possible realisations of a two-stage trial, it is biased conditional on reaching stage 2. In order to provide an estimator that can always be applied in a general two-stage trial, Pepe *et al.* suggest augmenting the UMVCUE with the stage 1 MLE when the trial does indeed stop at stage 1. Although this ‘composite’ estimator is generally biased, Pepe *et al.* show that it can actually have a smaller MSE than the UMVUE for small true values of *p*. We will apply the same composite estimator in place of the standard UMVCUE in future sections where appropriate and will refer to it as the ‘c-UMVCUE’.

## 3. Numerical example

**Figure 2:**
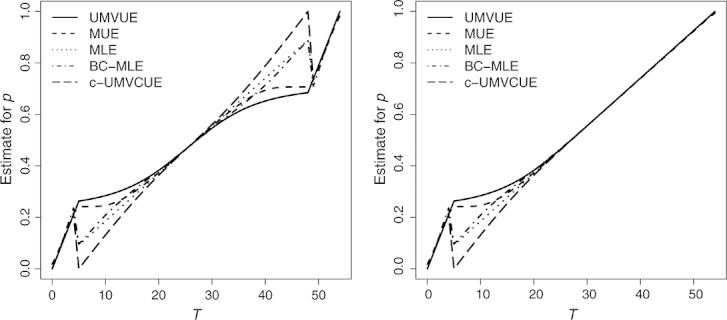
Value of estimators as a function of *T* for a Shuster-type trial (left) and a Simon-type trial (right). Note that the composite estimator c-UMVCUE is used here.

To demonstrate the implementation of these estimators for a specific two-stage Shuster-type trial, we initially take the data example in [1], giving design parameters Ω ≡ (*l*_1_,*u*_1_,*u*_2_,*n*_1_,*n*_2_) = (4,14,16,19,54). These values were chosen to provide at least 90% power to detect a response probability of 0.4 in the experimental treatment with a maximum type I error rate of 5%, assuming a known response probability for standard treatment of 0.2. Figure 2 (left) shows all estimators’ values across the entire sampling space of *T* for this design. Figure 2 (right) shows the estimators’ values for a Simon-type design, achieved by setting *u*_1_ = 20. The greatest difference between the estimators occurs at *T* = *l*_1_ + 1 for the Shuster and Simon designs and at *T* = *n*_2_ − *n*_1_ + *u*_1_ for the Shuster design. Only the MUE and UMVUE are monotonically increasing functions of *T*.

### 3.1. Estimator performance

**Figure 3:**
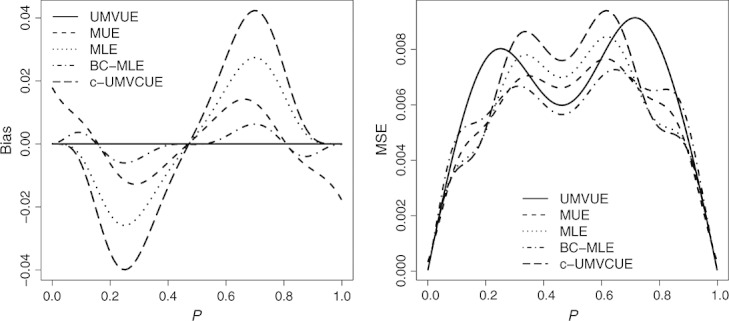
Bias (left) and MSE (right) for all estimators over the entire parameter space for the Shuster-type two-stage design. Note that the composite estimator c-UMVCUE is used here.

In order to assess the performance of each estimator in terms of bias and MSE, we need to assign the correct probability to each specific realisation of *T*. For Shuster-type trials, we modify an expression in [8] to give the probability that *T* = *t* given *p* using *T*-mapping as shown in Table I. Figure 3 shows the results for all values of *p* in (0,1). Note that the bias of all estimators is symmetrical about *p* = 0.5. By definition, the UMVUE is unbiased across the two-stage design, whereas all other estimators exhibit some bias. The least biased of these is the BC-MLE.

For values of *p* in approximately (0.35, 0.65), the BC-MLE has the smallest MSE, whereas for values of *p* in (0, 0.2) and (0.8, 1), it is the c-UMVCUE that performs best. The fact that there are two regions of *p* for which the c-UMVCUE works very well is counterintuitive but not wholly unexpected, given previous observations with respect to Simon-type trials [10].

**Figure 4:**
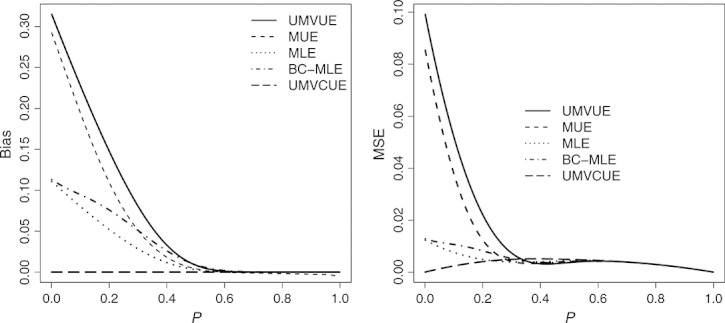
Conditional bias (left) and MSE (right) for all estimators over *T* = {*l*_1_ + 1,*n*_2_} for the Simon-type design. Note that the standard UMVCUE is used here

To illustrate estimator performance for a Simon-type design, we follow the advice of Pepe *et al.* and evaluate the bias and MSE of each estimator conditional on reaching stage 2. The *T*-map for this conditional probability is shown in Table I, and the results are shown in Figure 4. By definition, the UMVCUE is unbiased, whereas other methods can exhibit substantial (conditional) bias. For example, when *p* is close to 0, the bias of the UMVUE and MLE is close to 0.3. Of course, the smaller the value of *p*, the less chance there is of reaching stage 2 in practice. The UMVCUE generally performs very well in terms of MSE, too, but is marginally bettered by the other estimators for *p* in the region (0.3, 0.6). This indicates that even though the MUE, MLE, BC-MLE and UMVUE are being applied here out of their original context, they may still have some utility.

## 4. Optimal designs incorporating estimator performance

A two-stage design specification Ω is often chosen in order to minimise the expected sample size at some value of *p* [5, 6, 11]. Although there is considerable merit in this, optimal designs that focus on a single criterion can have poor properties when evaluated using other criteria of interest. For this reason, Jung *et al.* [2] proposed searching for a set of admissible Simon-type designs that balance two criteria of interest: the expected sample size under *p* = *p*_0_ and the maximum sample size *n*_2_. It was shown that the admissible set contains designs that have expected sample sizes close to Simon's optimal design but with a considerably lower *n*_2_.

We can extend the idea of Jung *et al.* to different criteria as well as a greater number. For example, Mander *et al.* [3] add the expected sample size under *p* = *p*_1_ as a third criterion. The method for finding admissible designs for an arbitrary set of criteria is straightforward in the case of a two-stage design with binary end points. Firstly, all possible candidate designs with correct type I error rate and power are evaluated at the criteria. As the number of parameters is low and all parameters are integer-valued, this is not generally computationally intensive. Secondly, all designs that minimise the weighted sum of the chosen criteria for some set of weights are found (by searching over a fine grid of weights). These are the admissible designs.

We propose finding admissible pairings of design and estimator that incorporate both expected sample size (ESS) and estimator performance. Admissible pairings are with respect to three summary statistics, namely, ESS, absolute bias and MSE. Because the three quantities are on different scales, we re-scale each so that they take values in the interval [0, 1]. The re-scaled value of a quantity, *x*, for a particular design is as follows:


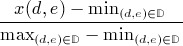


where *d* indexes the design, *e* the estimator, and 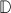 is the set of feasible pairings (i.e. pairings with designs that have the correct type I error rate and power). We always include a uniformly unbiased estimator (i.e. UMVUE or UMVCUE depending on scenario), so that the minimum absolute bias is fixed at 0. For each weighting of the three criteria, a pairing of a two-stage design together with an estimator, (*d*,*e*), is found, which minimises the weighted sum:


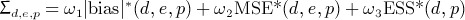
(4)

where * indicates that the statistic is its re-scaled version, *d* is the two-stage design, *e* indicates the estimator used and *p* is the parameter value at which the summary statistics are evaluated. The *ω* parameters are constrained to be non-negative and sum to 1. Some further points are as follows: ESS is independent of the estimator used, different definitions of bias and MSE are possible, and the value of the response probability *p* used to evaluate each summary statistic can also be different. Some specific scenarios we will explore are the following:

1. ESS is evaluated at *p* = *p*_0_, whereas the bias and MSE are evaluated at *p* = *p*_1_.
2. All three quantities are evaluated under a Uniform (*p*_0_,*p*_1_) distribution.
3. Bias and MSE are evaluated across all possible trial realisations.
4. Bias and MSE are evaluated for trials reaching stage 2 only.

The first possibility reflects the situation where ESS under the null is of most interest, as advocated by [6], whereas the bias and MSE are of interest for larger values of *p*. This could be because the results may be used to plan a larger study, so the estimation properties are of more interest for values of *p* that are more likely to result in trial success. The second possibility reflects the situation where little is known about the effectiveness of the treatment, so the average properties over a plausible range of response probabilities are of interest. The third and fourth possibilities are assumed for Shuster-type and Simon-type trials, respectively.

### 4.1. Application to Shuster-type trials

**Figure 5:**
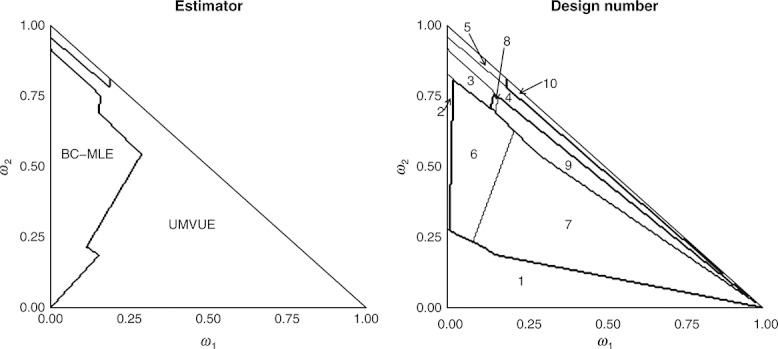
Left: The values of *ω*_1_ and *ω*_2_ supported by the two chosen estimators. Right: The values of *ω*_1_ versus *ω*_2_ as a function of the 10 distinct admissible design and analysis pairings (for a Shuster-type trial).

In this section, we examine properties of admissible pairings for Shuster-type trials. Table II shows the set of admissible pairings for *p*_0_ = 0.2, *p*_1_ = 0.4, *α* = 0.1, *β* = 0.2 under scenario 1. The table shows a wide range of designs, although perhaps surprisingly few considering that there are three admissibility criteria and five different estimators under consideration. Interestingly, only two of the five estimators are utilised within the admissible pairings. Figure 5 shows the values of *ω*_1_ and *ω*_2_ from Equation (4) supporting each estimator (left) and each design from Table II (right). The UMVUE is generally chosen when more of the weight is on the bias of the design, whereas the bias-corrected MLE is generally chosen when more of the weight is on the MSE. This observation agrees with the results from the specific design featured in Figure 3, where for *p* = 0.4, the BC-MLE had the lowest MSE and the second lowest absolute bias, whereas the UMVUE had the lowest bias and the second lowest MSE. Design 4 is an exception to the rule.

**Table II:** Admissible pairings for a Shuster-type trial; *p*_0_ = 0.2, *p*_1_ = 0.4, *α* = 0.1, *β* = 0.2

Design (*n*_1_, *l*_1_, *u*_1_, *n*_2_, *u*_2_)	ESS (*p*_0_)	Estimator	| Bias (*p*_1_) |	MSE (*p*_1_)
1. (11, 2, 5, 31, 10)	17.6	BC-MLE	8.25E − 03	1.79E − 02
1. (11, 2, 5, 31, 10)	17.6	UMVUE	0	2.10E − 02
2. (13, 3, 11, 34, 10)	18.3	BC-MLE	3.47E − 03	1.08E − 02
3. (16, 4, 13, 33, 10)	19.4	BC-MLE	2.61E − 03	9.83E − 03
4. (18, 4, 18, 31, 10)	21.7	UMVUE	0	8.98E − 03
5. (17, 3, 13, 30, 10)	22.9	BC-MLE	1.51E − 03	8.77E − 03
6. (13, 3, 12, 34, 10)	18.3	BC-MLE	2.95E − 03	1.08E − 02
7. (13, 3, 13, 34, 10)	18.3	BC-MLE	2.79E − 03	1.08E − 02
7. (13, 3, 13, 34, 10)	18.3	UMVUE	0	1.15E − 02
8. (16, 4, 14, 33, 10)	19.4	BC-MLE	2.21E − 03	9.87E − 03
9. (16, 4, 16, 33, 10)	19.4	UMVUE	0	1.01E − 02
10. (17, 3, 17, 30, 10)	22.9	UMVUE	0	8.79E − 03

Design numbers match with those in Figure 5 (right).

An interesting observation is that the same design can appear in multiple pairings, with different estimators. Pairing 1 from Table II has the lowest ESS at *p* = *p*_0_, so if *ω*_3_ is sufficiently high it will be the chosen design no matter the values of *ω*_1_ or *ω*_2_. Other pairings of note are ‘5’, which minimises the MSE, and ‘6’ or ‘7’, which are optimal when bias, MSE and ESS are given relatively equal weighting. Apart from the first design listed, all the designs have a high efficacy boundary in the first stage—this is likely just due to the fact that the expected sample size is evaluated at *p* = *p*_0_.

Amongst all feasible designs, the correlation between the ESS and bias of the BC-MLE is − 0.38; the correlation between its ESS and MSE is − 0.43 and the correlation between its bias and MSE is 0.7. We observe the same qualitative pattern for all estimators, but the precise correlation values vary. Designs that have lower ESS generally perform worse in terms of estimator properties. Additionally, designs for which the bias is large will generally have larger values of MSE as well. Of course, for any estimator that is uniformly unbiased (under the stated criteria), all paired correlations involving bias are undefined.

Table III shows results for the second scenario, where instead of evaluating the quantities under a single value of *p*, they are integrated over a U (*p*_0_,*p*_1_) distribution. The design parameters are *p*_0_ = 0.2, *p*_1_ = 0.4, *α* = 0.1, *β* = 0.2. In comparison with Table II, which showed results for the same design parameters, there are a greater number of admissible designs. We could explain this by the MUE being admissible as well as the bias-corrected MLE and UMVUE. Another observation of interest is that the designs that put a high weight on ESS are notably different from the ones in Table II. This is because ESS is evaluated over the interval [*p*_0_,*p*_1_] rather than just at *p*_0_. The difference in designs is consistent with the properties of different optimal designs as shown, for example, in [5].

**Table III:** Admissible pairings for a Shuster-type trial; *p*_0_ = 0.2, *p*_1_ = 0.4, *α* = 0.1, *β* = 0.2 with quantities of interest evaluated assuming a U(*p*_0_,*p*_1_) distribution

Design (*n*_1_, *l*_1_, *u*_1_, *n*_2_, *u*_2_)	ESS	Estimator	| Bias |	MSE
(15, 3, 6, 27, 9)	19.6	BC-MLE	3.75E − 03	1.26E − 02
(15, 3, 6, 27, 9)	19.6	UMVUE	0	1.34E − 02
(17, 4, 7, 25, 8)	19.7	MUE	3.03E − 03	1.14E − 02
(17, 4, 7, 25, 8)	19.7	UMVUE	0	1.19E − 02
(19, 5, 7, 28, 9)	20.5	BC-MLE	1.43E − 03	1.06E − 02
(19, 5, 7, 28, 9)	20.5	MUE	3.55E − 03	1.04E − 02
(19, 5, 7, 28, 9)	20.5	UMVUE	0	1.09E − 02
(20, 5, 7, 26, 9)	21.0	BC-MLE	1.02E − 03	1.02E − 02
(20, 5, 7, 26, 9)	21.0	MUE	3.36E − 03	9.95E − 03
(20, 5, 7, 26, 9)	21.0	UMVUE	0	1.03E − 02
(22, 5, 8, 24, 8)	22.6	UMVUE	0	9.30E − 03
(20, 4, 20, 24, 8)	22.9	MUE	3.83E − 04	8.95E − 03
(22, 5, 22, 24, 8)	23.3	MUE	1.52E − 03	8.65E − 03
(22, 5, 22, 24, 8)	23.3	UMVUE	0	8.91E − 03
(20, 0, 14, 24, 8)	24.0	BC-MLE	2.46E − 04	8.54E − 03
(20, 0, 15, 24, 8)	24.0	BC-MLE	1.80E − 04	8.56E − 03
(22, 0, 16, 24, 8)	24.0	BC-MLE	9.04E − 05	8.58E − 03
(22, 0, 22, 24, 8)	24.0	MUE	2.67E − 03	8.37E − 03
(22, 0, 22, 24, 8)	24.0	UMVUE	0	8.61E − 03

### 4.2. Application to a Simon-type trial

We next examined admissible pairings for Simon-type trials. Estimation of *p* was only considered if the trial reached stage 2, and therefore we used conditional definitions of bias and MSE. The scenario examined was the same as that in Table II, except that early stopping for efficacy was not possible. That is, the expected sample size was evaluated at *p* = *p*_0_, and the conditional estimation properties were evaluated at *p* = *p*_1_. The admissible designs and analysis pairings, together with their properties, are shown in Table IV. Note that we revert to using the standard UMVCUE in this example.

**Table IV:** Admissible pairings for a Simon-type trial; *p*_0_ = 0.2, *p*_1_ = 0.4, *α* = 0.1, *β* = 0.2

Design (*n*_1_, *l*_1_, *n*_2_, *u*_2_)	ESS (*p*_0_)	Estimator	| Bias (*p*_1_) |	MSE (*p*_1_)
(13, 3, 34, 9)	18.3	UMVCUE	0	8.23E − 03
(13, 3, 34, 9)	18.3	UMVUE	0.041	5.07E − 03
(13, 3, 34, 9)	18.3	MUE	0.024	5.38E − 03
(13, 3, 34, 9)	18.3	MLE	0.016	6.32E − 03
(10, 2, 38, 10)	19.0	MUE	0.022	4.99E − 03
(10, 2, 38, 10)	19.0	UMVCUE	0	6.98E − 03
(7, 1, 37, 10)	19.7	UMVCUE	0	6.95E − 03

Compared with Table II, there are fewer unique designs within the admissible pairings (three in total), and the range of expected sample sizes is much narrower, but we utilise four out of the five estimators examined. This indicates that there are near-optimal designs that have good (conditional) estimation properties. The only estimator not present is the BC-MLE. The UMVCUE appears multiple times, as it is unbiased, although it also has the largest MSE. If one puts a low weight on the bias, then the MUE is a good alternative estimator. Interestingly the UMVUE has the highest conditional bias and the lowest conditional MSE for the design (13, 3, 34, 9). A noteworthy observation is that the traditional method of carrying out two-stage cancer trials, that is, the null-optimal design coupled with the MLE, is one of the admissible pairings. Also, the potential reward of deviating from the optimal design varies considerably with the estimator used. For example, using the UMVCUE, one can reduce the MSE by around 23 *%* for a 4 *%* increase in ESS. On the other hand, if one uses the MUE, the potential gain in estimation properties is much smaller (an 8 *%* decrease in bias and a 7 *%* decrease in MSE).

## 5. Discussion

Much attention has focused on identifying clinical trial designs that minimise the expected (or maximum) sample size, and for good reason. However, these ‘optimal’ designs can inadvertently lead to response probability estimates with poor properties. In this paper, we argue that although the sample size will always be the main priority, optimal design and analysis plans can easily be sought that trade off small amounts of expected sample size for expected estimator performance. For example, in Table III, the (15,3,6,27,9) design is optimal in terms of ESS, but the near-optimal design (17,4,7,25,8) has around a 10*%* lower MSE for each estimator considered for only a 0.5*%* higher ESS. To some, this might be a trade-off worth making. Software for implementing this methodology can be found at http://www.mrc-bsu.cam.ac.uk/software.html.

Our results have shown that the bias-corrected MLE and UMVUE estimators consistently appear as admissible. For Shuster-type trials, the UMVUE will often be desirable if one places more weight on bias than MSE and the bias-corrected MLE if vice versa. For Simon-type trials, the UMVCUE performs very well, and the median unbiased estimator also becomes admissible in some scenarios and generally appears to have higher bias and lower MSE compared with the bias-corrected MLE.

The particular design and analysis plan identified by our method will clearly be sensitive to the values of response probability *p* at which the quantities of interest are evaluated. We demonstrated approaches using single values of *p* and also considered scenarios where *p* varied uniformly within a certain range to demonstrate how additional uncertainty could be incorporated. Extensions to more sophisticated distributions for *p* are obvious. The chosen value or distribution of *p* could be inferred directly from previously run phase II trials of similar regimens or from a previous phase I trial of the same regimen that recorded efficacy data.

We chose to search for optimal designs that minimise ESS, bias and MSE as a way to illustrate the general method. Others may prefer to incorporate different estimation summaries such as variance or Pitman closeness; if used, the latter measure might well favour estimators that are median unbiased [12]. One may also wish to incorporate the ability of an estimation routine to furnish accurate confidence intervals as well as point estimates. The MUE and UMVUE are certainly attractive from this point of view as the *p*-value function defined by the FM ordering in Section 2 can be used directly to obtain confidence intervals, whereas only bootstrap approximations have been so far developed for the UMVCUE [10, 13]. Regardless of the estimation criteria used or the particular design under investigation, we recommend considering all estimators in the optimisation procedure. From Figures 3 and 4, one can see that it is not impossible for an estimator that is being applied in a setting far from its original purpose to offer potential utility.

As further work, we also plan to extend this methodology to multi-stage trials and to trials with continuous end points by incorporating estimator performance into the admissible designs methodology of Wason *et al.* [14]. A continuous end point makes the optimisation problem harder because one cannot enumerate all feasible designs so easily.
